# Uncovering biomarker genes with enriched classification potential from Hallmark gene sets

**DOI:** 10.1038/s41598-019-46059-1

**Published:** 2019-07-05

**Authors:** Colin A. Targonski, Courtney A. Shearer, Benjamin T. Shealy, Melissa C. Smith, F. Alex Feltus

**Affiliations:** 1 Clemson University, Department of Electrical and Computer Engineering, Clemson, SC 29634 USA; 2 Clemson University, Department of Genetics and Biochemistry, Clemson, SC 29634 USA; 3 Clemson University, Center for Human Genetics, Clemson, SC 29634 USA; 4 Clemson University, Biomedical Data Science and Informatics, Clemson, SC 29634 USA

**Keywords:** Machine learning, Cancer genomics

## Abstract

Given the complex relationship between gene expression and phenotypic outcomes, computationally efficient approaches are needed to sift through large high-dimensional datasets in order to identify biologically relevant biomarkers. In this report, we describe a method of identifying the most salient biomarker genes in a dataset, which we call “candidate genes”, by evaluating the ability of gene combinations to classify samples from a dataset, which we call “classification potential”. Our algorithm, Gene Oracle, uses a neural network to test user defined gene sets for polygenic classification potential and then uses a combinatorial approach to further decompose selected gene sets into candidate and non-candidate biomarker genes. We tested this algorithm on curated gene sets from the Molecular Signatures Database (MSigDB) quantified in RNAseq gene expression matrices obtained from The Cancer Genome Atlas (TCGA) and Genotype-Tissue Expression (GTEx) data repositories. First, we identified which MSigDB Hallmark subsets have significant classification potential for both the TCGA and GTEx datasets. Then, we identified the most discriminatory candidate biomarker genes in each Hallmark gene set and provide evidence that the improved biomarker potential of these genes may be due to reduced functional complexity.

## Introduction

### Biomarkers

Biomarkers are indicators of biological processes^1^. Since the advent of molecular biology, thousands of molecular biomarkers (DNA, epiDNA, RNA, protein, metabolite) have been discovered and linked to a broad range of clinical conditions. Any accepted biomarker would have undergone statistical analysis and peer review to verify its diagnostic, prognostic, and predictive power. However, for many biomarkers, it is not always clear if their classification potential is a function of causality or simply a tight association with a phenotype. Further, the combination of individual biomarkers into multivariate molecular signatures confound the issue by mixing potentially causal and associative molecular events. While this complexity is challenging, untangling the actual molecular control mechanisms captured within molecular signatures is a requirement to transition from grouping biological processes into truly understanding them.

As perspective on genetic complexity, a recent annotation of the human genome (GENCODE)^2^ describes 58,381 human genes transcribing 203,805 RNA transcripts with protein coding transcripts yielding 61,132 distinct protein translations. Each of these features is a potential biomarker for a given phenotype. Within this biological complexity are functional genetic subsystems (polygenic molecular signatures) that control most human phenotypes, and it is a difficult problem to circumscribe phenotypically relevant molecular signatures and their alleles. One method to place biomarkers into functional context is to link genes to pathway databases such as REACTOME^3^ and KEGG^4^. Gene interaction sub-networks are another source of potentially co-functional physical, temporal, and genetic molecular interactions described in databases like BioGrid^5^ and IntAct^6^. A powerful, broad ranging, and popular collection of curated gene sets controlling known biological processes is the The Molecular Signatures Database (MSigDB), developed as a source of co-functionally enriched genes for biological condition association via Gene Set Enrichment Analysis^7^. Since these gene sets are enriched for common biological function, it is plausible that the combined information in a given gene set, captured via the quantification of gene output features (e.g. RNA gene expression quantification), will have a higher likelihood of being a causal molecular signature controlling the phenotype.

### Classification approaches

One method to discover biomarkers is to classify categorical biological conditions using quantitative gene expression measurements as features in neural network models. Deep learning^8^, a term encompassing a broad class of neural network techniques, has had enormous success in image^9^ and natural language processing^10^ applications. Deep learning classification systems have made significant progress in recent years, predominantly due to the work of Krizhevsky *et al*.^11^ in which a deep convolutional neural network significantly outperformed previous state of the art techniques for image classification. This work opened the door for a variety of deep neural networks to be applied to a diverse range of applications; from anomaly detection^12^ to tumor classification^13^. Because of its ability to extract high level abstractions from the raw features of very large datasets, deep learning fits the mold for biological dataset analysis^14^. While there exists a plethora of machine learning approaches to analyze data such as gene expression, neural networks in particular have provided increasingly impressive results in bioinformatics applications^15^.

### A priori feature selection

High throughput gene measurement techniques like RNA sequencing (RNAseq)^16^ capture the global gene expression profile for thousands of genes in a biological sample at high resolution. For a given sample, a RNAseq gene profile can be coupled with metadata describing the biological sample that may include experimental conditions, tissue source, disease state (if any), and clinical variables describing the sampled individual. These feature sets can be used to cluster and classify samples into knowledge-dependent groups mined from the metadata. For example, the global gene expression profiles for thousands of human tumors from The Cancer Genome Atlas (TCGA)^17^ have been used to sort tumors into clinically relevant subtypes and have identified specific molecular signatures associated with the tumor groups^18–20^. This top-down approach has yielded new and valuable knowledge of genes associated with tumor biology.

In this study, we implemented a bottom-up classification approach where we tested curated molecular signatures to classify biological samples using a deep learning model. Our reasoning was that if a set of genes known to have common biological function can classify biological samples into meaningful groups, then that gene set is a molecular signature biomarker that should be investigated more thoroughly by a domain expert. Thus our algorithm acts as a filter that reduces the search space of genes from tens of thousands to a handful. Herein, we describe our algorithm and its application to classify complex mixtures of normal human tissue from the Genotype-Tissue Expression (GTEx)^21^ dataset and abnormal tumors from the TCGA dataset using RNA expression profiles for MSigDB gene sets. Our algorithm is a GPU-enabled software package called Gene Oracle, which is available at https://github.com/SystemsGenetics/gene-oracle under the MIT open source license.

## Materials and Methods

### Molecular signature gene expression matrix preparation

RNAseq^16^ is a high-throughput DNA sequencing technique used to quantify RNA in a biological sample. RNA-seq datasets from normal human tissue samples (GTEx)^21^ were downloaded as a single RPKM unit gene expression matrix (GEM) from https://storage.googleapis.com/gtex analysis v7/rna seq data/GTEx Analysis 2016-01-15 v7 RNASeQCv1.1.8 gene tpm.gct.gz. RNA-seq datasets for human tumor samples from TCGA^17^ patients were downloaded in individual FPKM files in May 2018 using the GDC Data Transfer Tool (https://gdc.cancer.gov/access-data/gdc-data-transfer-tool). The RNAseq distributions of the GTEx and TCGA datasets can be found in Figs S1 and S2, located in the Supplemental Information section. The GTEx GEM contained a total of 11,688 samples across 56,202 genes representing 53 uniquely labeled tissue types with class distribution illustrated in Fig. S6. The TCGA GEM was collected from 11,092 patients across 60,483 genes representing 33 unique tumor types of distinct disease progression with class distribution as seen in Fig. S6. We downloaded 17,786 Broad Institute MSigDB gene lists^7^ from http://software.broadinstitute.org/gsea/msigdb/genesets.jsp?collection=H. For this study, we parsed the global GEMs for GTEx or TCGA into sub-GEMs for 50 molecular signatures called the Hallmark gene sets. Each MSigDB Hallmark gene set contained between 30 and 200 genes.

### Molecular signature classification

**Figure 1:**
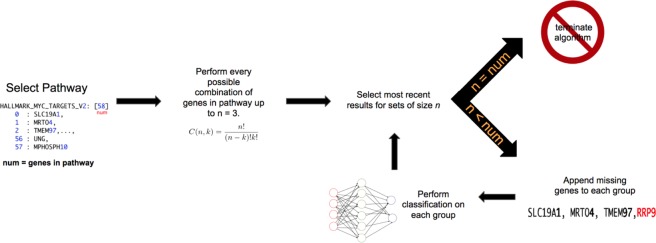
The Gene Oracle Algorithm.

Gene Oracle is designed to decompose a gene set into its most discriminatory features by iteratively appending genes to explore new combinations. The process consists of two phases. In phase I, a gene set is tested for significant classification potential relative to the same number of random gene features. In 143 phase II, the gene set undergoes a combinatorial decomposition in order to discover the most discriminatory genes in the set. The initial screening of gene sets is necessary due to the computational burden of the second phase. A graphical overview of the algorithm is shown in Fig. 1 and details of each phase are in the following subsections.

The “classification potential” of a gene set refers to the ability of the gene set to classify samples from a gene expression dataset. In this study we measured the classification potential of a gene set using a multilayer perceptron (MLP) neural network. The MLP consists of an input layer, three hidden layers, and a final softmax layer for classification. The input layer size is the size of the gene set, and the output layer size is the number of classes in the dataset. The hidden layer sizes are 512, 256, and 128 units respectively. Each hidden layer uses the rectified linear unit^22^ (ReLU) activation function. The MLP is initialized using Glorot (Xavier) initialization^23^ and trained using the Adam optimizer^24^ with a learning rate of 0.001. The MLP is trained on 70% of the dataset and then evaluated on the remaining 30% (the partition is determined randomly). The final classification potential is the average test accuracy from 10 train/test runs, each with a random train/test split (bootstrapping). The network was constructed in Python using Tensorflow^25^.

#### Phase I: Gene set classification screen

In order to identify which Hallmark gene sets were able to classify biological conditions, the GTEx or TCGA sub-GEMs for 50 Hallmark sets were screened for their classification potential based upon tissue (GTEx) or tumor (TCGA) categories. Each Hallmark set was evaluated using the MLP described previously. Additionally, for each Hallmark set, 50 random gene sets equal in size to the given Hallmark set were selected from all genes in the input dataset (TCGA or GTEx) and evaluated using the same MLP classifier. For example, the Hedgehog Signaling pathway contains 36 genes, so it was compared to 50 different sets of 36 random genes by classification potential. If the classification potential of a Hallmark set was higher than the average classification potential of its corresponding random sets with a statistical significance of *p* < 0.01 (using Student’s *t*-test), the set was considered for further analysis via combinatorial decomposition.

#### Phase II: Gene set combinatorial decomposition

For gene sets which exhibited a high classification potential, we developed a method for exploring the space of subsets within a gene set in order to identify the genes which contribute the most to classification potential. The total number of subsets of size *k* in a set of *n* genes is given by Eq. 1 (*n choose k*), and the total number of subsets in such a set is given by Eq. 2. Since this number grows rapidly with *n*, it is extremely computationally expensive to evaluate every possible subset within a gene set. Therefore, we only consider subsets of size *k* for *k* = 1, 2, 3 (note: we also refer to *k* as the “iteration”). This exhaustive method provides a repository of subsets, each of which have been evaluated for classification potential. From this point, a heuristic approach is taken to provide a comprehensive sampling of the subset space in a computationally tractable manner. As in phase I, all gene subsets are evaluated using the MLP classifier described previously.

1 $${C}_{k}^{n}=\frac{n!}{k!(n-k)!}$$

2 $${C}^{n}=\begin{array}{c}n\\ k=1\end{array}\,\frac{n!}{k!(n-k)!}$$

From the repository of evaluated subsets, the algorithm selects the subsets with the highest classification potential. These highest performing subsets are used to seed the next iteration of subsets by appending genes from the given Hallmark set. This procedure is repeated for each iteration – selecting the highest performing subsets, and using them to seed the next iteration – up to the size of the Hallmark set. In particular, given a Hallmark set of *n* genes, a list of *m*_*k*_ subsets of size *k*, and the *M* seed subsets taken from the *m*_*k*_ subsets, *n* − *k* subsets are created from each seed subset by appending each gene from the Hallmark set which is not already in the seed subset. Thus, *m*_*k*+1_ = *M* (*n* − *k*) new subsets of size *k* + 1 are created. Note that it is possible for duplicate subsets to be created; these duplicate subsets are removed. The remaining unique subsets are evaluated using the MLP, after which the *M* highest performing subsets are selected and used as the seed subsets for the next iteration, and so on. We used *M* = 60 for the experiments described in this paper.

This procedure is designed to reduce the number of subsets that must be evaluated by exploring only the “paths” of subsets which produce the best classification results. However, this criterion alone is biased towards the subsets which are selected early on. Therefore, in order to promote diversity among subsets, we introduce a second mechanism in which random subsets are also included as seed subsets. In particular, given *M* seed subsets, we also select *M/*2 *random* subsets to be seeds for the next iteration. Thus, the total number of subsets created at each iteration is given by Eq. 3.

3 $${m}_{k+1}=\frac{3}{2}M(n-k)$$

As an example, the Hedgehog Signaling Hallmark set contains 36 genes, which yielded ${C}_{3}^{36}$ = 7140 subsets of 3 genes. From this list we selected the *M* = 60 subsets with the highest classification accuracy, as well as $\tfrac{M}{2}=30\frac{M}{2}$ random subsets, as the seeds for the next iteration, which produced *m*_4_ = 90(36 − 3) = 2970 subsets of size 4. These subsets are evaluated using the MLP; this process continues up to *k* = *n*, the size of the Hallmark set. The output is a log of all the subsets that were created and their respective classification accuracies.

### Candidate gene selection

As mentioned previously, the goal of Gene Oracle is to identify the genes in a gene set which are most capable of classifying RNA-seq tissue samples. The iterative method described in Section 2.2.2 produces a frequency heatmap (see Results) which contains the frequency of each gene in all of the subsets of each iteration. Using this data, we determine a set of “candidate genes” as the genes with the highest aggregate frequency; that is, for each gene we compute the sum of the frequencies across all iterations, and we create a distribution of aggregate frequencies. Each gene whose aggregate frequency is at least one-half a standard deviation above the mean is included in the candidate set.

## Results

### Molecular signature screening

The Gene Oracle algorithm was evaluated for its effectiveness in uncovering the most salient molecular signatures from 50 Hallmark gene sets. The RNA expression values for both the GTEx and TCGA datasets were extracted from the master GEMs and used to classify the labeled subgroups using the MLP.

**Figure 2:**
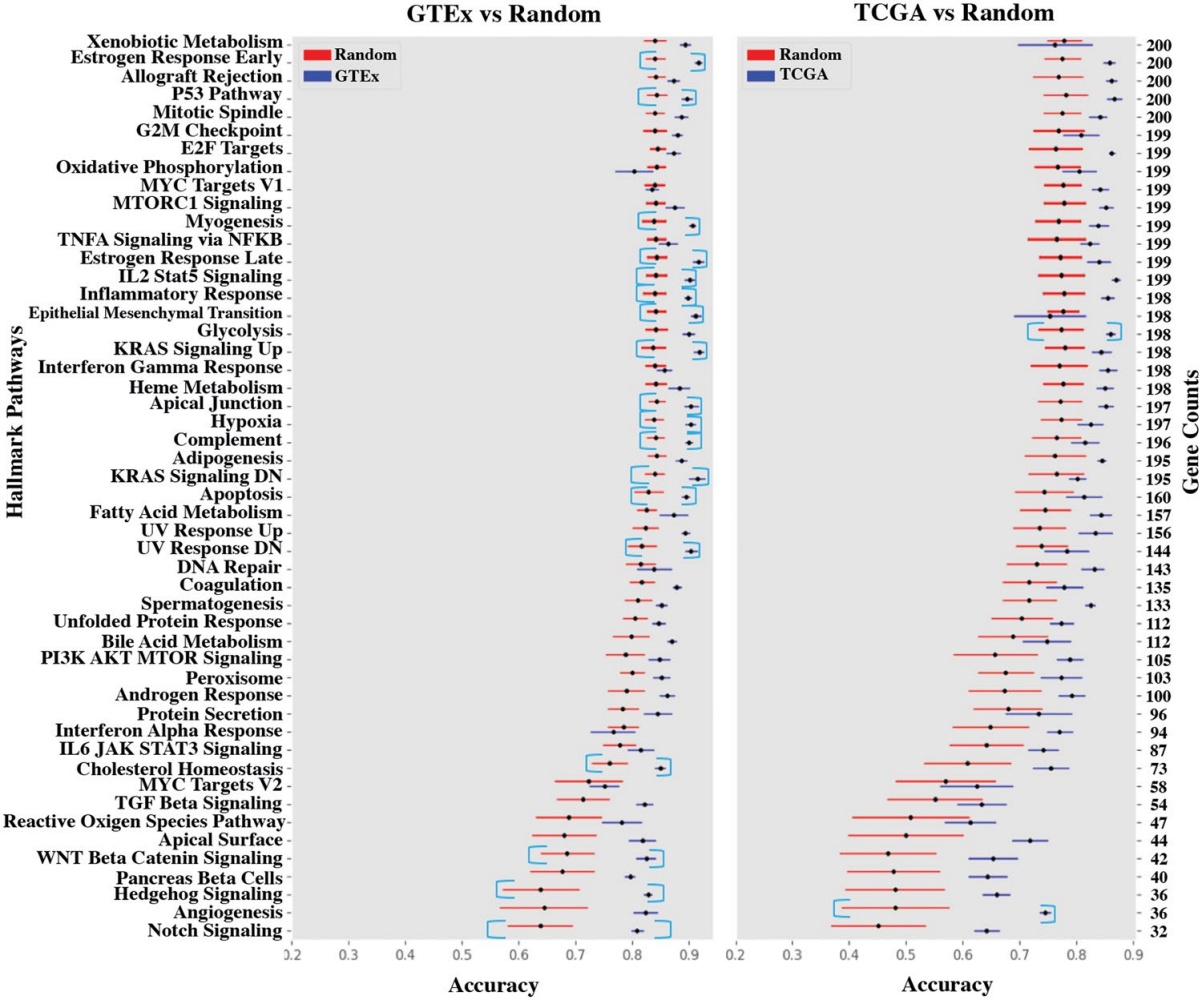
Molecular Signature Classification Screening. Plots depict the classification accuracy of each Hallmark set versus random sets of equal size in both GTEx and TCGA. The left y-axis displays the 50 Hallmark sets. The right y-axis displays the number of genes in each Hallmark set. The x-axis displays the accuracy score produced by the MLP. The light blue brackets denote Hallmark sets that exhibit higher classification accuracy over their random counterparts.

Each Hallmark set was compared to 50 random sets of equal size. The results for all 50 Hallmark sets on both the GTEx and TCGA datasets are shown in Fig. 2.

### Molecular signature subset analysis

Of the Hallmark sets that passed the screening (cf. light blue brackets in Fig. 2), the following sets were selected for full combinatorial analysis with Gene Oracle: Notch Signaling, Hedgehog Signaling, and Angiogenesis. These Hallmark sets were selected arbitrarily, as not all of the eligible Hallmark sets could be analyzed in phase II due to computational constraints. We also consider MYC Targets V2 as an example of a set which does not perform significantly better than its random counterparts. Using the combinatorial decomposition algorithm described previously, Gene Oracle identified genes which contributed the most to overall classification accuracy (candidate genes) for each Hallmark set.

**Figure 3:**
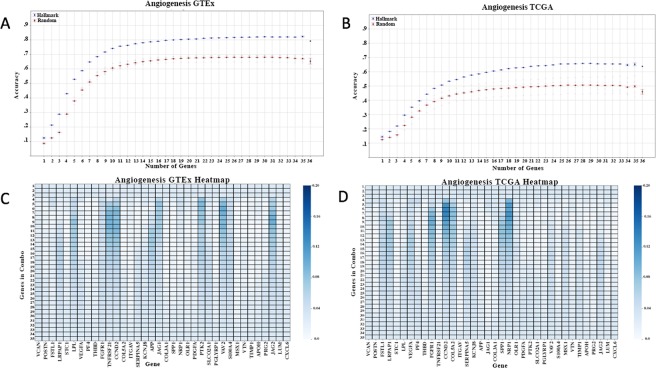
Combinatorial Analysis of Angiogenesis. (**A**,**B**) Accuracy plots of subsets from Angiogenesis versus 5 random sets of equal size for GTEx (left) and TCGA (right). In each plot, the darkest random line represents the average of the 5 random experiments, while the faded lines represent the results of individual random experiments. (**C**,**D**) Heatmaps depicting the frequency of genes in the subsets that were generated at each iteration. Each row is an iteration and each column is a gene from the Hallmark Angiogenesis set. Darker colors correspond to higher frequencies.

Figure 3A,B show the classification accuracy at each iteration of the Gene Oracle algorithm for the Hallmark Angiogenesis set. Figure 3C,D are heatmaps which show the number of times a gene occurs in a subset at a given iteration of the combinatorial analysis. For example, at iteration 7 in Fig. 3C, the genes that occur most frequently are TNFRSF21, CCND2, PTK2, and VAV2, because these genes have the darkest entries in the heatmap at that iteration. The values in the first three rows of the heatmap are constant across all genes because every possible combination of genes is evaluated for the first three iterations of the analysis. As Gene Oracle progresses further into the analysis, the distribution of gene frequencies becomes varied. Combinatorial analyses for the other selected Hallmark sets are provided in Supplemental Figs S3, S4 and S5.

Using the heatmaps as a guide (e.g. Fig. 3), candidate genes were determined for Hedgehog Signaling, Angiogenesis, Notch Signaling, and MYC Targets V2, for both GTEx and TCGA (Tables 1 and 2, respectively). Genes with an aggregate frequency of at least one-half the standard deviation above the mean were selected as candidate genes; the remaining genes in the Hallmark set were marked as “non-candidate” genes.

**Table 1:** Gene Oracle Candidate Genes for GTEx Dataset.

Hallmark Set	Candidate Genes
Hedgehog Signaling	SHH, UNC5C, HEY2, L1CAM, NKX6-1, CRMP1, CELSR1, CNTFR, CDK5R1, VLDLR, DPYSL2
Angiogenesis	LRPAP1, LPL, FGFR1, TNFRSF21, CCND2, APP, JAG1, PTK2, VAV2, S100A4, JAG2
Notch Signaling	FZD1, PSEN2, FZD7, CUL1, DTX4, HEYL, ARRB1, PRKCA, ST3GAL6, FBXW11
MYC Targets V2	SLC19A1, TMEM97, NDUFAF4, MYC, SRM, GNL3, TBRG4, NPM1, CBX3, CDK4, MAP3K6, LAS1L, PUS1, SLC29A2, DCTPP1, SORD

**Table 2:** Gene Oracle Candidate Genes for TCGA Dataset.

Hallmark Set	Candidate Genes
Hedgehog Signaling	NRP1, HEY2, TLE3, TLE1, ETS2, VEGFA, CELSR1, CDK5R1, OPHN1
Angiogenesis	FSTL1, LRPAP1, VEGFA, FGFR1, CCND2, COL5A2, SERPINA5, SPP1, NRP1
Notch Signaling	FZD1, FZD7, FZD5, NOTCH1, DTX4, ARRB1, PRKCA, ST3GAL6
MYC Targets V2	SLC19A1, RRP9, IPO4, MYC, SRM, GNL3, RABEPK, PLK1, PRMT3, MAP3K6, SLC29A2, DCTPP1, SORD, UNG, MPHOSPH10

**Figure 4:**
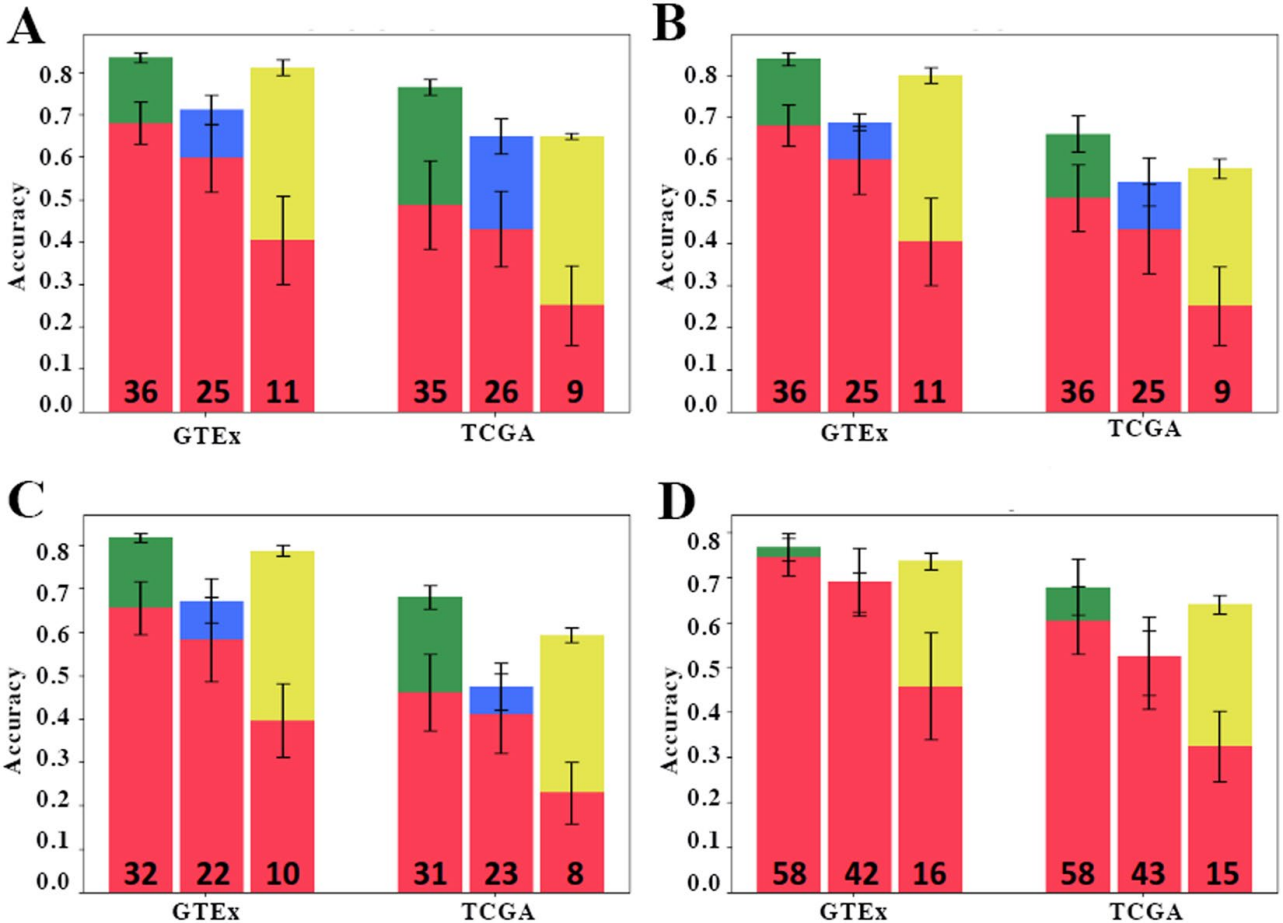
Classification potential for decomposed gene sets. Classification accuracies for the full Hallmark gene sets (green) are compared to accuracies of the candidate genes identified by the combinatorial decomposition algorithm (yellow), non-candidate genes (blue), and random genes (red). The performance analysis for (**A**) Hedgehog Signaling, (**B**) Angiogenesis, (**C**) Notch Signaling, and (**D**) MYC Targets V2 Hallmark sets are shown for both GTEx and TCGA datasets. The number at the bottom of each bar denotes the size of the gene sets for that result.

To verify the classification potential of the candidate genes, we trained and evaluated the MLP classifier for each candidate set in Tables 1 and 2. Figure 4 shows, for each dataset and Hallmark set under consideration, the classification accuracy of (1) the original Hallmark set, (2) non-candidate set, and (3) candidate set. Additionally, each set was compared to 50 random sets of equal size, whose accuracies were averaged, in order to show the “relative” accuracy of each gene set. In each case, the difference in accuracy versus random is highest in the candidate set and lowest in the non-candidate set. In almost every case, the candidate set exhibits similar accuracy to its original Hallmark set, whereas the non-candidate set consistently exhibits lower accuracy compared to the original set.

### Gene ontology functional profiling

**Figure 5:**
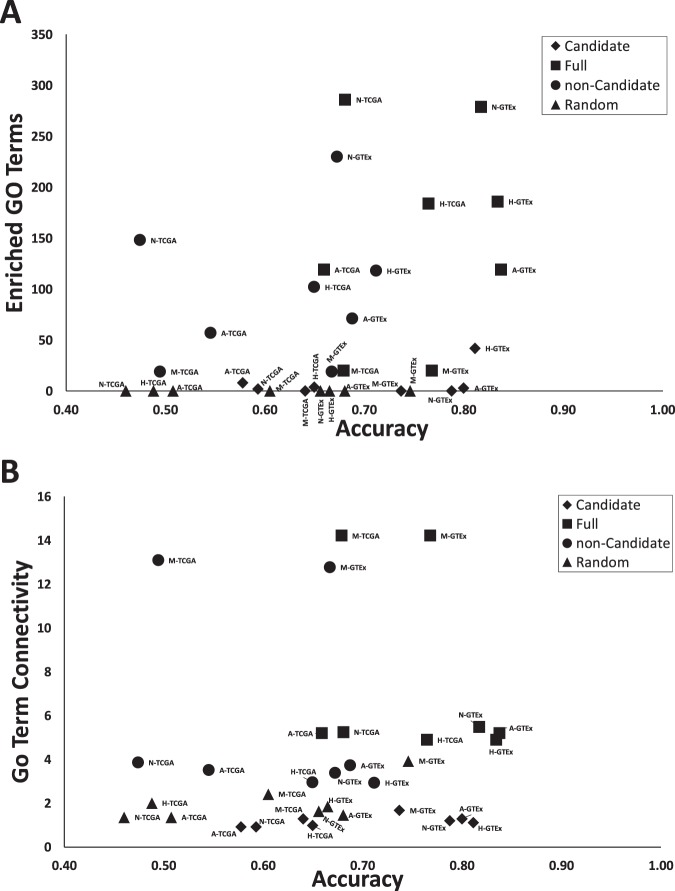
Functional Complexity Analysis. Gene Ontology (GO) term enrichment was performed for each full hallmark, candidate, non-candidate, or random gene set tested with TCGA or GTEx GEM where N = Notch signaling, H = Hedgehog signaling, A = Angiogenesis, M = MYC Targets V2. (**A**) The total number of enriched GO terms is shown (Adj. *p* < 0.001). (**B**) The average connectivity 〈k〉 of unique GO terms shared between genes in a set is shown.

To examine the functional complexity of each gene set, we performed a functional enrichment analysis of Gene Ontology (GO) terms using the ToppFunn webserver^26^. The Hallmark, candidate, non-candidate, and random sets were all analyzed. The number of enriched GO terms (q-value FDR B&Y adjusted *p* < 0.001) is shown in Fig. 5A. For each gene set, the full list of GO terms for each gene was found by looking up the gene in Ensembl Genes v24 using Biomart^27^ and aggregating its GO terms. For each gene set, the number of intersections between the GO term lists of the corresponding genes was taken as the number of edges, and the number of unique GO terms was taken as the number of nodes. The average connectivity score 〈k〉 is shown in Fig. 5B.

## Discussion

### Classification considerations

Gene Oracle leverages deep learning techniques to perform biological sample classification using multidimensional gene expression features. The MLP^28^ described in Section 2.2 is a commonly used model for classification tasks. The core advantage of a MLP lies in its layered structure and its use of nonlinear activation functions; the MLP can be composed of as many layers as necessary to capture the complexity of a dataset, and it can capture nonlinear relationships within the data. The layers of an MLP, once trained, transform the input data into a representation which is more suitable for classification. It should be noted that in our study we input RNAseq features into Gene Oracle, but any quantitative measurement of gene output would be acceptable input. Additionally, other machine learning models such as *k*-Nearest Neighbors, Support Vector Machines^29^, and Random Forests^30^ could have also been used in lieu of the MLP, and in the future we would like to repeat the experiments in this study with other classifiers in order to further validate our results.

Given the data-intensive nature of this analysis, it was imperative for the Gene Oracle algorithm to train models rapidly in order to minimize the overall run time, since a new model was trained and evaluated for each gene subset. The overall run time depends on several hyperparameters, most notably of which are the network size, the number of seed subsets for each iteration, and the size of the Hallmark set. Since Tensorflow^25^ has built-in support for GPU acceleration using NVIDIA’s CUDA and cuDNN libraries^31^, the MLP for a typical subset can be trained and evaluated in a few seconds on an NVIDIA P100 or V100 GPU. The faster that the model for a subset can be trained and evaluated, the more subsets that Gene Oracle can consider from the entire search space in a given time frame.

In order to determine a suitable network size for the MLP, we compared a variety of network architectures by varying both the number of hidden layers and the number of units per layer. We found that larger networks generally achieve higher accuracy at the cost of higher training time, which is consistent with the expected behavior of neural networks. We chose the 512 × 256 × 128 architecture for its ability to provide reasonably high classification accuracy with low training time, as our goal was not to simply maximize the classification accuracy of the MLP but to also provide a consistent metric by which to compare gene sets.

A key concept of our approach is what we refer to as “knowledge dependent feature selection” in which we only use gene sets for which there is accepted evidence for common biological function. The noteworthy performance of these curated sets is evident when comparing to random sets of equal size. For the large majority of Hallmark sets evaluated in this paper, the curated set of genes outperforms several randomly sampled sets of the same size. These results demonstrate the inherent classification potential of curated gene sets which are known to be involved in specific biological processes. Further, the algorithm runs faster in a reduced feature space.

### Reasons for misclassification

Sample misclassification can occur for a multitude of reasons. Dataset imbalance (i.e. uneven class sizes) can cause a neural network to become biased towards classes which contribute more training samples^32^ (see Fig. S6). When the set of features is reduced to a known Hallmark set and then to a subset, a large amount of information is lost simply from removing the majority of features, which leads to lower classification accuracy. Inherent gene expression noise and mislabeled samples could also lead to misclassification. These issues are compounded by variation in the RNA sequencing and processing techniques by the TCGA and GTEx data generator consortia. Further, the samples in the GTEx dataset are likely to be “cleaner” and tissue specific relative to the TCGA dataset, whose samples are composed of heterogenous tumor tissue. This provides a possible explanation as to why Hallmark sets generally classify samples from GTEx more effectively than from TCGA. Still, the classification accuracies are very high as seen in the confusion matrices (Supplemental Figs S7 and S8), ROC curves (Supplemental Figs S9 and S10), and precision-recall curves (Supplemental Figs S11 and S12).

### Molecular signatures as biomarkers

Gene Oracle identified specific Hallmark gene sets that significantly classify 53 “normal” GTEx samples and 33 “abnormal” TCGA tumor subtypes (Fig. 2). Furthermore, Gene Oracle identified subsets from selected Hallmark sets that classify nearly as well as the Hallmark sets themselves (Tables 1 and 2). Of the 50 Hallmark sets, 18 were significant classifiers of GTEx samples and 2 were significant classifiers of TCGA samples. Each of these signatures is a polygenic biomarker set determined, in our case, with RNA expression data.

Only one Hallmark gene set, Angiogenesis, was able to significantly classify both GTEx and TCGA samples. The Angiogenesis signature contains 36 genes that are involved in the development of blood vessels. These genes are critical to maintain enough blood supply so that normal tissue and tumors can survive^33^. Based on Fig. 3A,B, it is evident that nested candidate subsets of Angiogenesis genes consistently outperformed non-candidate genes. The GTEx gene expression output pattern pointed to 11/36 candidate genes (LRPAP1, LPL, FGFR1, TNFRSF21, CCND2, APP, JAG1, PTK2, VAV2, S100A4, JAG2) and the TCGA gene expression pattern pointed to 9/36 candidate genes (FSTL1, LRPAP1, VEGFA, FGFR1, CCND2, COL5A2, SERPINA5, SPP1, NRP1). Only 3/36 candidiate genes were shared between the two classification experiments: LRPAP1, FGFR1, CCND2. That is, even though the same Hallmark set classifies both sample groups, the nested subset of candidate genes is different for each case.

### Observed, background, and foreground biomarker classification potentials

In this study, we report the Gene Oracle classification accuracy, derived from gene expression data for a group of functionally related genes, as a measure of “observed classification potential” (OCP). Any gene set with an exceptionally high OCP can be considered a biomarker candidate gene set. However, the OCP of a gene set does not tell the full story. We previously showed in^34^ that random sets of approximately 100 or more genes, when embedded and visualized with t-SNE^35^, were able to discriminate five TCGA tumor subtypes. Furthermore, Macneil *et al*. performed gene set analysis based on a Support Vector Machine and found a correlation between gene set size and classification ability^36^. In this study, we observed this phenomenon in the full TCGA and GTEx datasets where the OCP of a Hallmark or random gene set tends to increase with the number of genes used in classification (Fig. 2). We define the classification accuracy of random genes as “background classification potential” (BCP). In other words, while some Hallmark gene sets have a high OCP, an equal number of random genes might exhibit an identical OCP which would indicate that the gene set, while discriminatory, is no better than random.

In Phase II of Gene Oracle, we identified the subset of Hallmark genes that contributed the most to OCP. These genes represent the best biomarker “candidate genes” which were separated from the non-candidate genes. Interestingly, every candidate gene subset identified by Gene Oracle for GTEx or TCGA exhibited a larger OCP relative to the BCP of random genes (Fig. 4). In fact, the candidate subset from the MYC Targets Hallmark set demonstrated significant OCP even though the full Hallmark set did not. We define the difference between gene set OCP and the BCP measured in random gene sets of equal size as the “foreground classification potential” (FCP) of the Hallmark gene set. Note that in all four cases, the discriminatory power of candidate versus non-candidate genes is not due to differences in gene expression variance between the two groups (F-test *p* < 0.05).

Any gene set with a high OCP contains condition-specific biomarkers. Genes with a high OCP and a high FCP (e.g. candidate genes) might also have a higher probability of encoding biological processes relevant to the condition being classified. What is the reason for variation in FCP in general and improved FCP for candidate genes in particular? One hypothesis is that some gene subsets have high FCP because they have more functional complexity than non-candidate subsets. To test this hypothesis, we examined the embedded molecular functions in all gene signatures through functional enrichment analysis as shown in Fig. 5. Each of the four Hallmark sets had more enriched GO terms and a higher GO term connectivity than random genes. After decomposing the gene set into candidate and non-candidate subsets, it was found that the candidate subset had fewer enriched GO terms and a lower GO term connectivity score than its non-candidate counterpart. Thus we were unable to reject the null hypothesis and it appears that a lack of functional complexity improves the FCP for candidate genes.

Why does reduced functional complexity lead to higher FCP? It may be that gene sets encoding more diverse and interconnected biological function could affect multiple biological processes driving classifier conditions and thus have less discriminatory power. When functional complexity is reduced, as seen in the candidate genes, more selective functions improve FCP. This could also explain BCP because the more genes you select, the more random discrete functions are encoded in the gene set leading to higher BCP. However, it is the selective functions in the non-random candidate genes that unmask the FCP diluted by the more functionally complex non-candidate genes.

## Conclusions

We introduce Gene Oracle, a hybrid machine learning and combinatorial approach used for discovering potential biomarkers with high classification potential (observed and foreground). The first phase of screening gene sets is not limited to the Hallmark sets considered in this paper; any curated gene set can be used as input to the algorithm. Furthermore, our method of selecting seed subsets during the second phase is a naive approach to a difficult problem, and many alternative approaches remain to be tested. Our initial experiments were focused on tackling this problem – that is, to select subsets in a way that maximizes classification accuracy while also covering as much of the search space as possible. After performing subset analysis for a few Hallmark sets, further exploration of the candidate and non-candidate subsets through their GO terms provided some explanation for the disparity in accuracy between them. We found that increasing the number of genes used as input features to a model can increase classification accuracy. However, using more genes can obscure the few biomarkers (candidate genes) which cover fewer biological functions yet provide nearly equivalent classification accuracy. Therefore, although it is easy to improve classification potential by random feature selection, more attention should be given to foreground classification – and how to distinguish it from background classification – in order to discover the predictors of biological processes with more specificity.

## Supplementary information

Dataset 1

## Data Availability

All raw data is available from public repositories as described in the Materials & Methods. Gene Oracle source code is available at https://github.com/SystemsGenetics/gene-oracle under the MIT open source license.
