# Supplementary material for: Uncovering biomarker genes with enriched classification potential from Hallmark gene sets
Source: Sci Rep. 2019 Jul 5;9:9747. doi: 10.1038/s41598-019-46059-1 (PMC6611793; doi:10.1038/s41598-019-46059-1)
Supplement: Supplementary file 1 — Dataset 1 [file 41598_2019_46059_MOESM1_ESM.pdf]

Supplemental Dataset 1

Uncovering biomarker genes with enriched classification potential from Hallmark gene sets

Colin Targonski, Courtney Shearer, Ben Shealy, Melissa C. Smith, and F. Alex Feltus

Supporting information

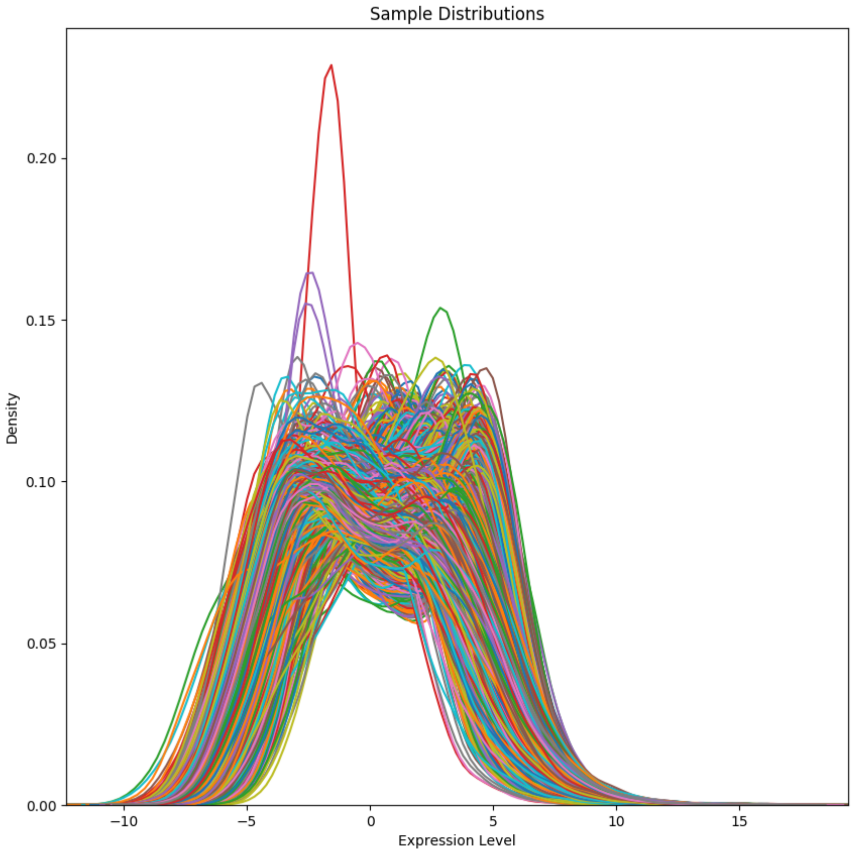

Fig S1. GTEx Density Plot. This plot depicts the densities of the GTEx dataset after performing a log2 transformation.

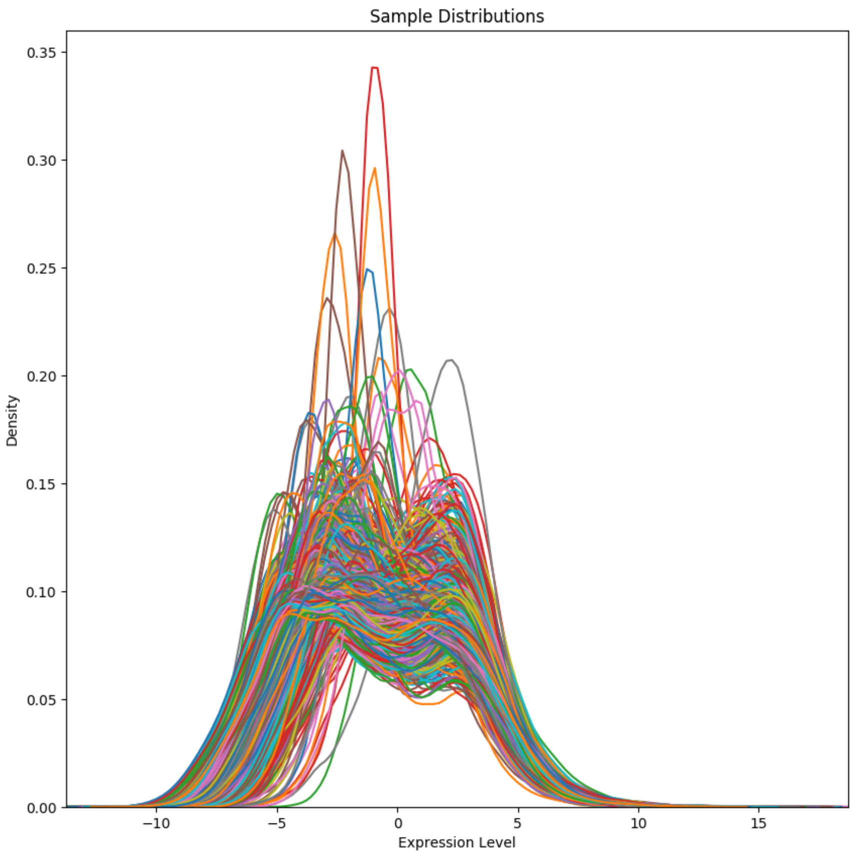

Fig S2. TCGA Density Plot. This plot depicts the densities of the TCGA dataset after performing a log2 transformation.

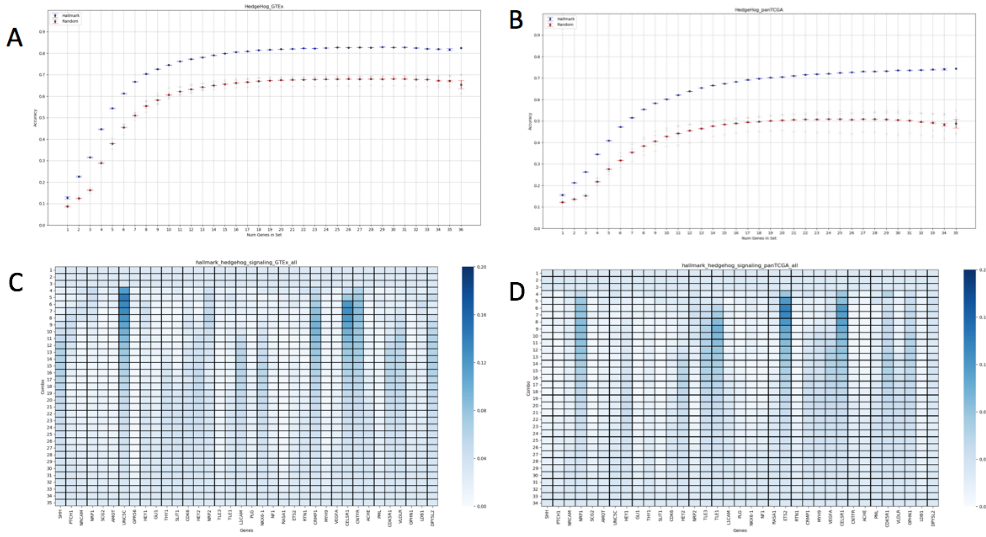

Fig S3. Combinatorial Analysis of HedgeHog Signaling.

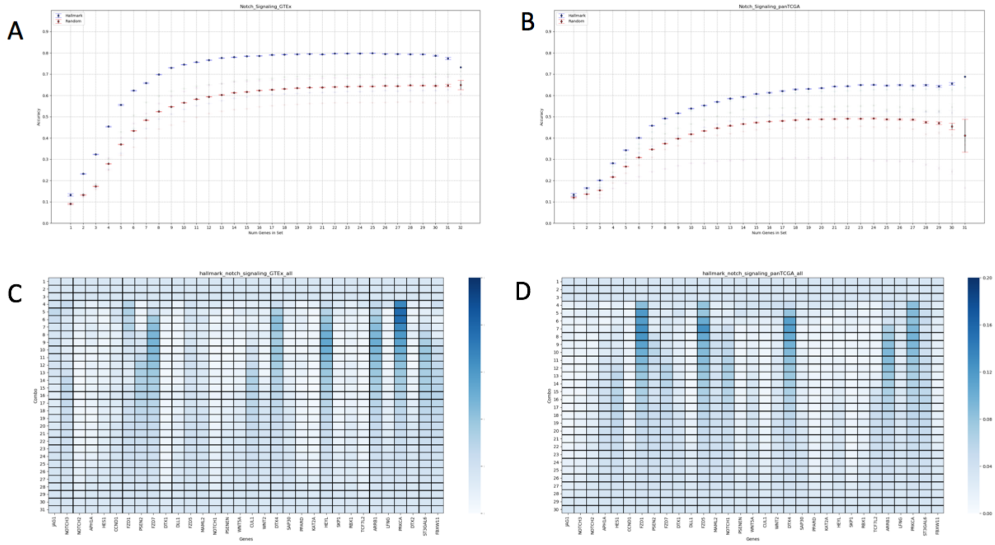

Fig S4. Combinatorial Analysis of Notch Signaling.

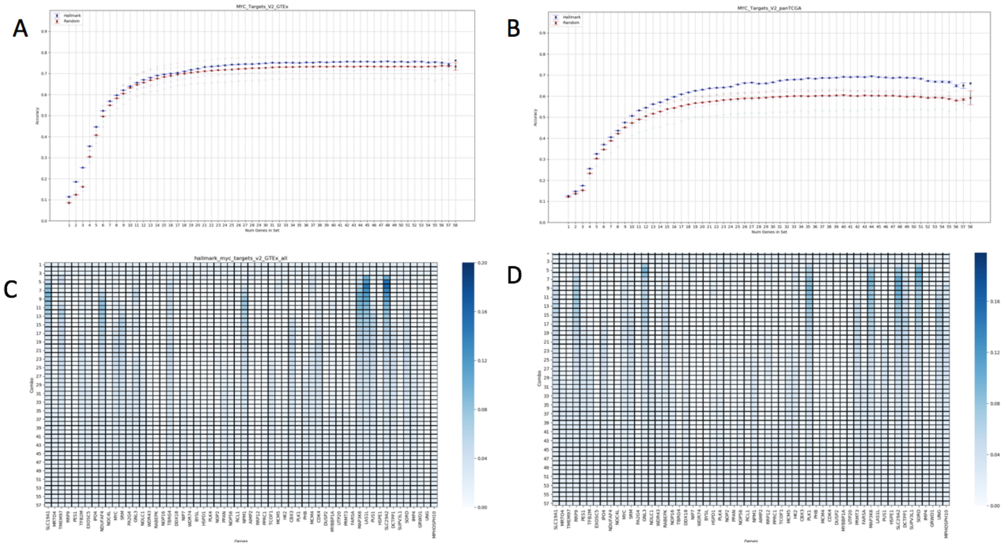

Fig S5. Combinatorial Analysis of MYC Target V2

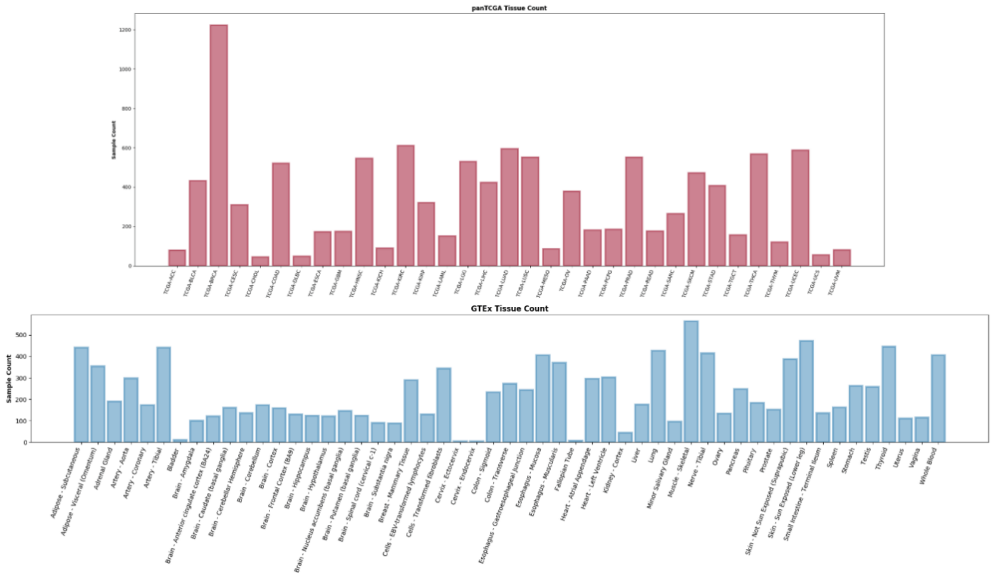

Fig S6. Biological categories in TCGA and GTEx datasets.

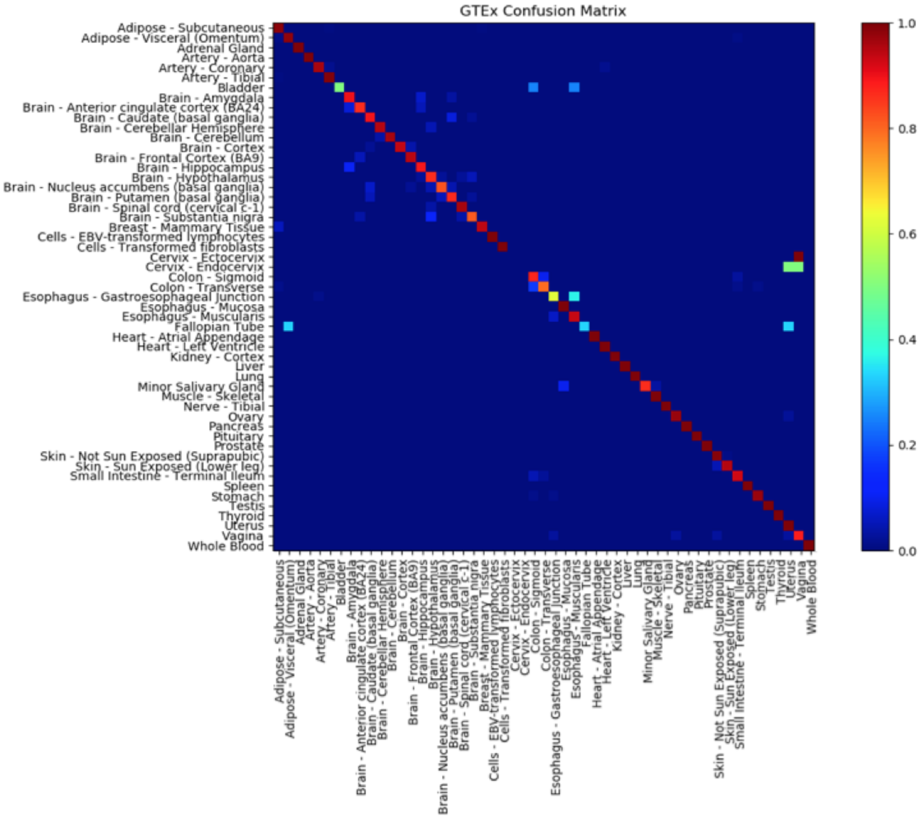

Fig S7. GTEx sensitivity and specificity analysis with a Confusion Matrix. A confusion matrix visualizes the true/false positives and true/false negatives of a classifier as a heatmap. A classifier that has no error would have red in the diagonal and blue everywhere else. This confusion matrix demonstrates the viability of tissue classification on the GTEx dataset using all genes.

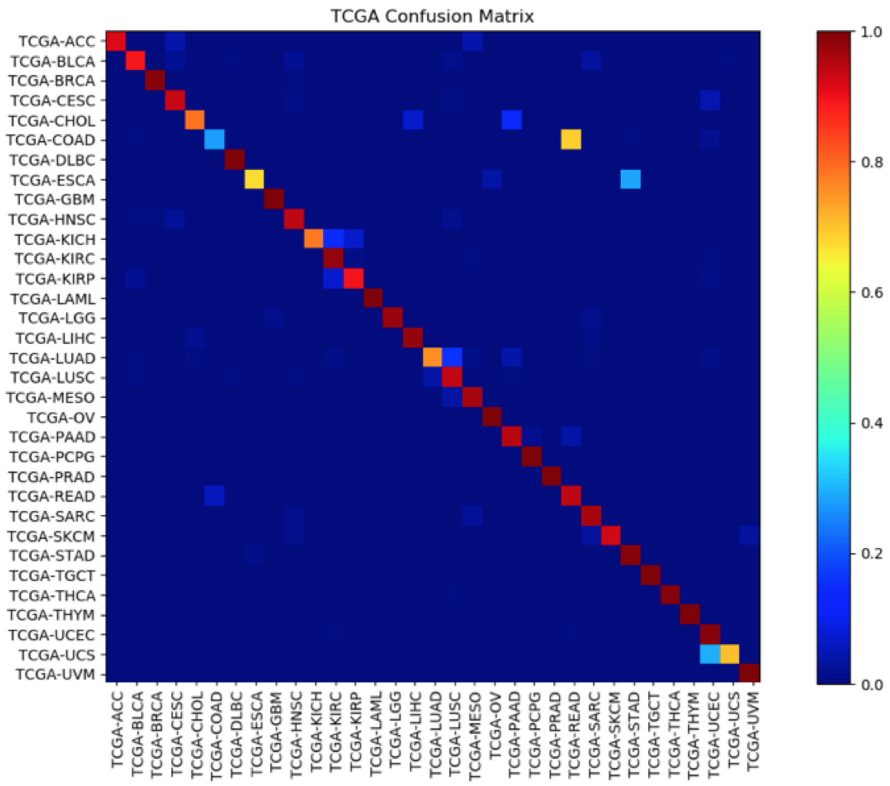

Fig S8. TCGA sensitivity and specificity analysis with a Confusion Matrix. A confusion matrix visualizes the true/false positives and true/false negatives of a classifier as a heatmap. A classifier that has no error would have red in the diagonal and blue everywhere else. This confusion matrix demonstrates the viability of tumor classification on the TCGA dataset using all genes.

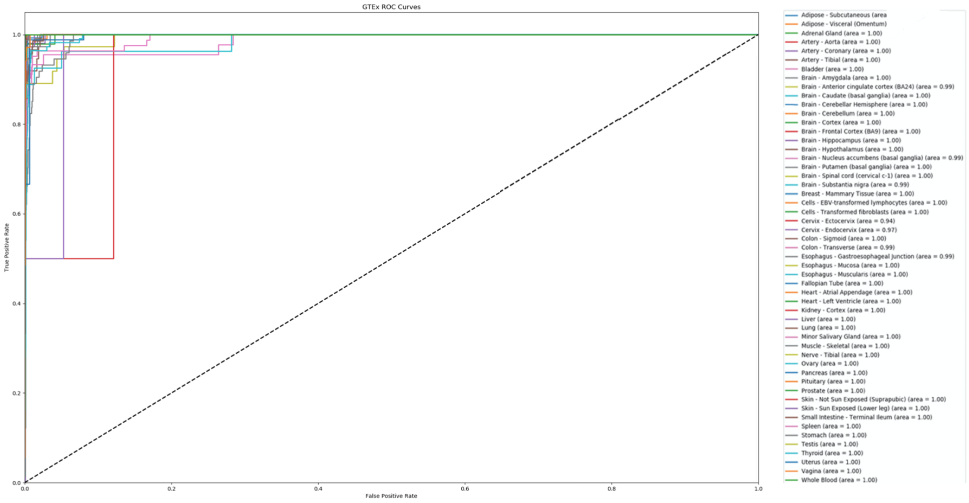

Fig S9. GTEx sensitivity and specificity analysis with a ROC Curve. The ROC curve, plots the True Positive Rate (TPR) on the x-axis by the False Positive Rate (FPR) on the y-axis across all tissue types.

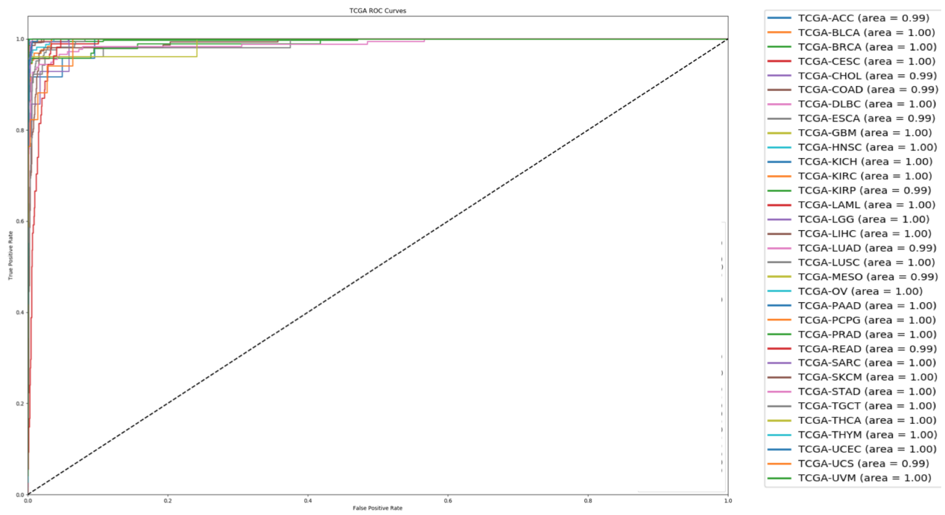

Fig S10. TCGA sensitivity and specificity analysis with a ROC Curve. The ROC curve, plots the True Positive Rate (TPR) on the x-axis by the False Positive Rate (FPR) on the y-axis across all cancer tumor types.

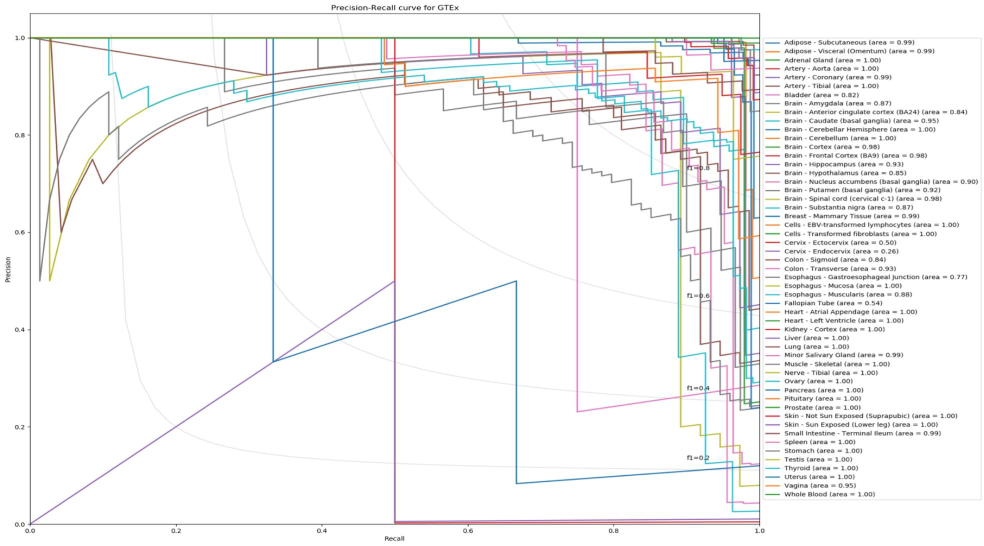

Fig S11. GTEx sensitivity and specificity analysis with a Precision Recall Curve. The Precision Recall Curve plots the precision of the classifier on the x-axis and the accuracy along the y-axis. F1 score curves are present for comparison.

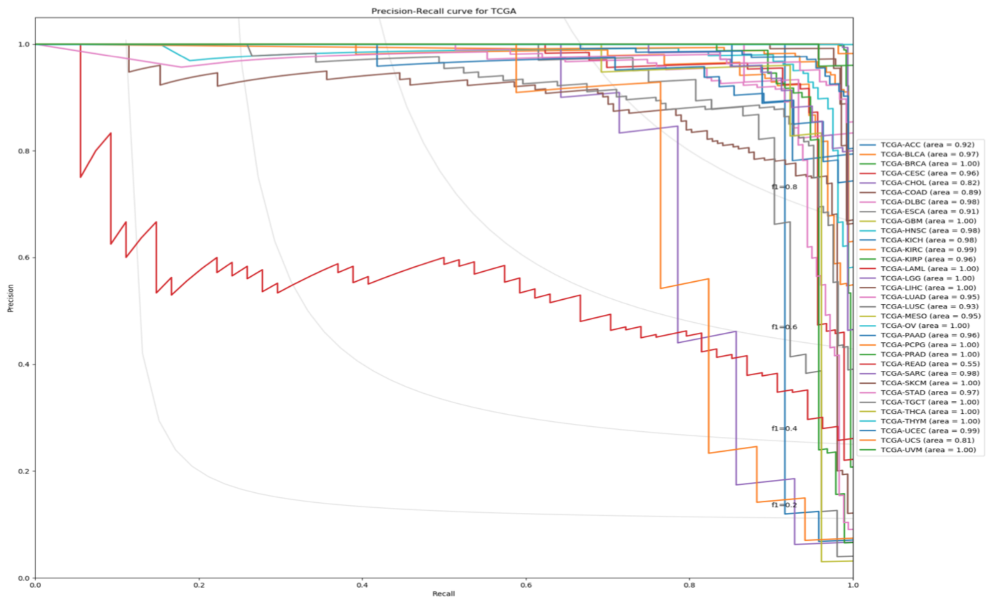

Fig S12. TCGA sensitivity and specificity analysis with a Precision Recall Curve. The Precision Recall Curve plots the precision of the classifier on the x-axis and the accuracy along the y-axis. F1 score curves are present for comparison.
